# 152. Use of Antimicrobials among Suspected COVID-19 Patients at Selected 12 Hospitals in Bangladesh: Findings from the First Wave of COVID-19 Pandemic

**DOI:** 10.1093/ofid/ofab466.354

**Published:** 2021-12-04

**Authors:** Syeda Mah-E-Muneer, Md Zakiul Hassan, Md Abdullah Al Jubayer Biswas, Zubair Akhtar, Pritimoy Das, Fahmida Rahman, Md Ariful Islam, Fahmida Chowdhury

**Affiliations:** icddr,b, Dhaka, Dhaka, Bangladesh

## Abstract

**Background:**

Antimicrobials are empirically used in COVID-19 patients resulting in inappropriate stewardship and increased antimicrobial resistance. Our objective was to assess antimicrobial use among suspected COVID-19 in-patients while waiting for the COVID-19 test report.

**Methods:**

From March to August 2020, we collected data from in-patients of 12 tertiary-level hospitals across Bangladesh. We identified suspected COVID-19 patients; collected information on antimicrobial received within 24 h before and on hospitalization; and tested nasopharyngeal swab for SARS-CoV-2 using rRT-PCR. We used descriptive statistics and a regression model for data analysis.

**Results:**

Among 1188 suspected COVID-19 patients, the median age was 34 years (IQR:2–56), 69% were male, 40% had comorbidities, 53% required oxygen, and 1% required ICU or ventilation support after admission. Antibiotics were used in 92% of patients, 47% within 24 h before, and 89% on admission. Patients also received antiviral, mostly favipiravir (1%) and antiparasitic drugs particularly ivermectin (3%). Third-generation cephalosporin use was the highest (708;60%), followed by macrolide (481;40%), and the majority (853;78%) who took antibiotics were SARS-CoV-2 negative. On admission, 77% mild and 94% moderately ill patients received antibiotics. Before admission, 3% patients had two antibiotics, and on admission, 27% received two to four classes of antibiotics at the same time. According to WHO AWaRe classification, the Watch group antibiotics were mostly used before (43%) as well as on admission (80%). Reserve group antibiotic particularly linezolid was used in 1% patients includes mild cases on admission. Antibiotic use on admission was higher among severely ill patients (AOR = 11.7;95%CI:4.5–30.1) and those who received antibiotics within 24 h before hospital admission (AOR = 1.6;95%CI:1.0–2.5).

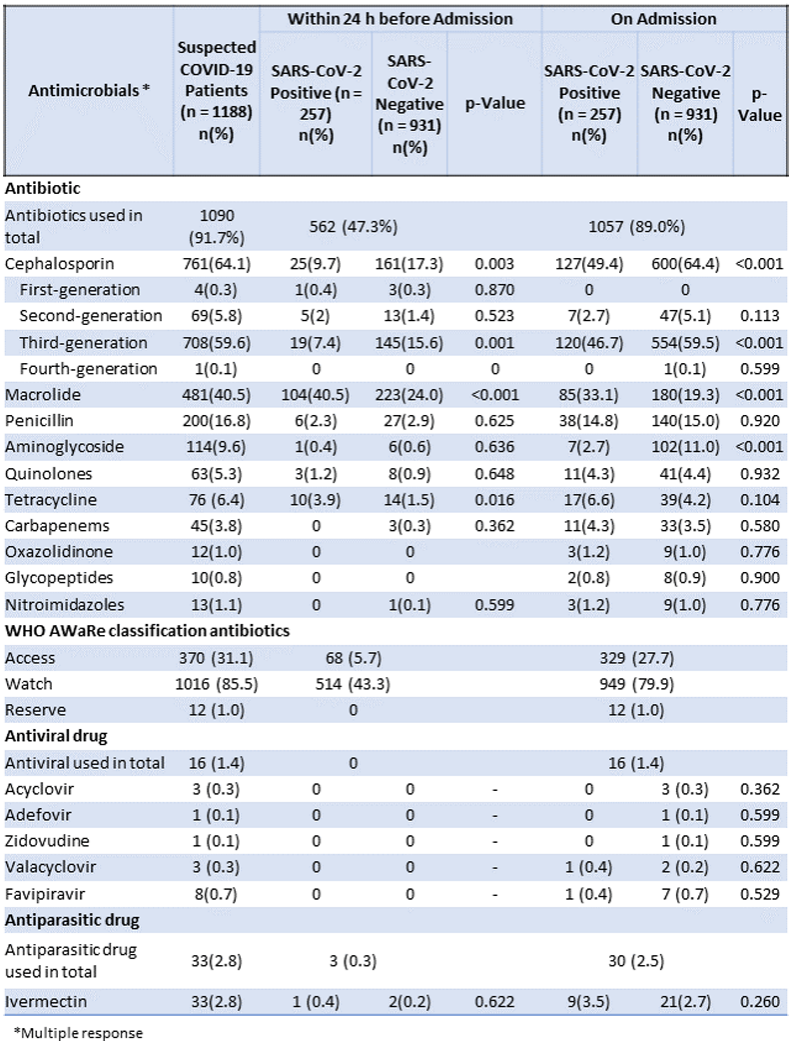

Antimicrobials used among suspected COVID-19 patients and SARS-CoV-2 positive and negative patients 24 h before and on hospital admission at 12 selected hospitals in Bangladesh, March–August 2020

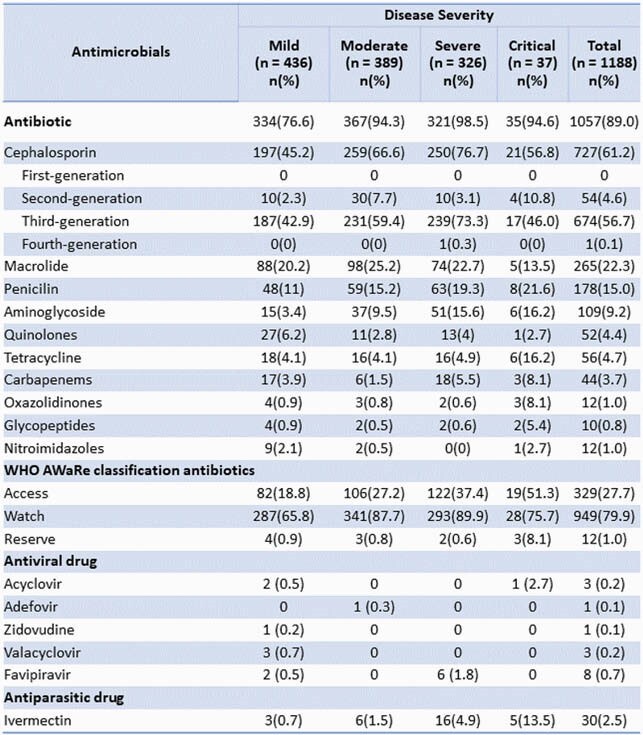

Antimicrobials used on admission among suspected COVID-19 patients according to disease severity at 12 selected hospitals in Bangladesh, March–August 2020

**Conclusion:**

Antimicrobial use was highly prevalent among suspected COVID-19 in-patients in Bangladesh. Initiating treatment with Watch group antibiotics like third-generation cephalosporin and azithromycin among mild to moderately ill patients were common. Promoting antimicrobial stewardship with monitoring is essential to prevent blanket antibiotic use, thereby mitigating antimicrobial resistance.

**Disclosures:**

**All Authors**: No reported disclosures

